# Adherence and intensity in multimodal lifestyle-based interventions for cognitive decline prevention: state-of-the-art and future directions

**DOI:** 10.1186/s13195-025-01691-0

**Published:** 2025-03-17

**Authors:** Natalia Soldevila-Domenech, Amaia Ayala-Garcia, Mariagnese Barbera, Jenni Lehtisalo, Laura Forcano, Ana Diaz-Ponce, Marissa Zwan, Wiesje M. van der Flier, Tiia Ngandu, Miia Kivipelto, Alina Solomon, Rafael de la Torre

**Affiliations:** 1 Integrative Pharmacology and Systems Neuroscience Research Group, Neuroscience Research Program, Hospital del Mar Research Institute, Dr. Aiguader 88, Barcelona, 08003 Spain; 2 Barcelonaβeta Brain Research Center (BBRC), Pasqual Maragall Foundation, Wellington 30, Barcelona, 08003 Spain; 3 Department of Neurology, Institute of Clinical Medicine, University of Eastern Finland, Yliopistonranta 1C, Kuopio, 70211 Finland; 4 The Ageing Epidemiology Research Unit, School of Public Health, Imperial College London, Charing Cross Hospital, St Dunstan’s Road, London, W6 8RP UK; 5 Population Health Unit, Finnish Institute for Health and Welfare, Mannerheimintie 166, P.O. Box 30, Helsinki, Finland; 6 CIBER de Fisiopatología de la Obesidad y Nutrición, Instituto de Salud Carlos III, Av. Monforte de Lemos 3-5, Madrid, 28029 Spain; 7 Alzheimer Europe, Sennengerbierg Nidderaanwen, Luxembourg City, 1736 Luxembourg; 8 Alzheimer Center, Department of Neurology, Neuroscience Campus Amsterdam, VU University Medical Center, De Boelelaan 1117, Amsterdam, 1081 HV Netherlands; 9 Division of Clinical Geriatrics, Center for Alzheimer Research, Department of Neurobiology, Care Sciences and Society, Karolinska Institute, Karolinska Vägen 37A, Solna, 171 64 Sweden; 10 Medical Unit Aging, Theme Inflammation and Aging, Karolinska University Hospital, Stockholm, D1: 04, 171 76 Sweden; 11 Institute of Public Health and Clinical Nutrition, University of Eastern Finland, Yliopistonrinne 3, Kuopio, FI-70211 Finland; 12 Department of Medicine and Life Sciences, Universitat Pompeu Fabra, Dr Aiguader 80, Barcelona, 08003 Spain; 13 Neurosciences Research Program, Hospital del Mar Research Institute (HMRI), Dr Aiguader 88, Barcelona, 08003 Spain

**Keywords:** Adherence, Intensity, Lifestyle intervention, Multimodal intervention, Alzheimer’s disease, Prevention; review

## Abstract

Preventing dementia and Alzheimer’s disease (AD) is a global priority. Multimodal interventions targeting several risk factors and disease mechanisms simultaneously are currently being tested worldwide under the World-Wide FINGERS (WW-FINGERS) network of clinical trials. Adherence to these interventions is crucial for their success, yet there is significant heterogeneity in adherence reporting across studies, hindering the understanding of adherence barriers and facilitators. This article is a narrative review of available evidence from multimodal dementia prevention trials. A literature search was conducted using medical databases (MEDLINE via PubMed and SCOPUS) to select relevant studies: nonpharmacological multimodal interventions (i.e., combining three or more intervention domains), targeting individuals without dementia, and using changes in cognitive performance and/or incident mild cognitive impairment or dementia as primary outcomes. Based on the findings, we propose future adherence reporting to encompass both participation (average attendance to each intervention component) and lifestyle change using dementia risk scores (e.g., the LIBRA index). Moreover, we provide an estimation of the expected intensity of multimodal interventions, defined as the ratio of the expected dose (i.e., the overall amount of the intervention offered specified in the trial protocol) to duration (in months). Adjusting the expected dose by average adherence enables estimation of the observed dose and intensity, which could be informative for identifying optimal dosage thresholds that maximize cognitive benefits across different populations. Finally, this article provides an overview of the determinants of adherence to multimodal interventions, emphasizing the need for improved adherence reporting to inform the design and implementation of precision prevention interventions.

**Supplementary Information:**

The online version contains supplementary material available at 10.1186/s13195-025-01691-0.

## Background

Alzheimer’s disease (AD), the most prevalent cause of dementia, develops over a long preclinical period and its progression is associated with modifiable lifestyle factors [1, 2]. This offers a window of opportunity for testing early preventive measures. In the last decade, there has been a shift toward multimodal lifestyle-based interventions for dementia prevention (e.g., combining diet, physical activity, cognitive training, vascular risk monitoring, or social interaction), due to the multifactorial nature of this condition [3]. In contrast to interventions targeting one risk factor alone, multimodal interventions target multiple risk factors simultaneously and are expected to generate additive or synergistic preventive effects. However, there is still limited evidence on the effectiveness of multimodal interventions for the prevention of cognitive decline [4]. Additionally, uncertainties persist regarding barriers and facilitators of adherence and response to multimodal interventions, modes of intervention delivery, and the intervention intensity (dose, duration, and adherence) required to influence cognitive performance [5]. Addressing these questions is crucial for advancing and optimizing multimodal interventions for dementia prevention, both at the individual (i.e., personalized prevention), and population levels (i.e., precision prevention) [6, 7].

The effectiveness of a next generation of precision prevention interventions for cognitive decline will rely on how effectively preventive programs are provided to populations of interest, their ability to adhere to such interventions, and the ability of healthcare providers to monitor adherence and adjust the intervention as needed. Adherence is recognized as the strongest predictor of intervention success [8]. However, there is a paucity of studies addressing the determinants of adherence to multimodal interventions [9–14]. This gap in evidence is, in part, attributed to the absence of a gold standard definition of adherence to these complex multimodal interventions. Although a single definition may not universally apply to all studies, the development of consensus-based recommendations for measuring and reporting adherence to multimodal interventions could enhance consistency across studies and thus establish global standards for conducting comparative effectiveness research. This is particularly important in the framework of international collaborative networks conducting multimodal intervention trials aimed at preventing cognitive decline, e.g., the World-Wide FINGERS (WW-FINGERS) network [15]. Harmonizing measures of adherence to multimodal interventions will facilitate pooled analyses, which are crucial for providing robust evidence about adherence profiles and progressing in AD prevention research.

The aim of this narrative review is to provide an overview of the available evidence on adherence and efficacy from multimodal dementia prevention trials.

## Main text

### Methodology

An English-language literature search was conducted using medical databases (MEDLINE via PubMed and SCOPUS, through November 29th, 2024) and keywords such as “multidomain”, “intervention”, “dementia”, “prevention” and “cognitive decline”. Additional studies were identified through the reference lists of selected publications and the researchers’ expertise on WW-FINGERS studies. The search strategy, screening process, and data selection adhered to PRISMA guidelines [16]. The following criteria were used to select relevant studies, including both randomized controlled trials (RCTs) and protocols: nonpharmacological multimodal interventions (defined as combining three or more intervention domains) with a duration of at least 6 months, a target population including individuals without dementia at baseline, and cognitive performance and/or incident mild cognitive impairment (MCI) or dementia as primary or secondary outcomes. This review was not registered in the International Prospective Register of Systematic Reviews (PROSPERO).

During the screening process, two independent reviewers (NS-D and AA-G) assessed eligibility based on titles and abstracts. Data extraction for the narrative review was conducted by one researcher (NS-D), capturing details on study design, multimodal intervention characteristics (e.g., dose, duration, adherence), and primary outcome measures. Quality assessment of the studies included was not conducted.

The database search identified 417 unique articles, with an additional 13 articles retrieved from other sources (Supplementary Fig. 1). After screening, 45 articles were selected for full-text review of clinical trials. Of these, 24 completed clinical trials met the inclusion criteria for analysis, and were distributed geographically as follows: 12 from Asia, 10 from Europe, and 2 from America. Among the excluded articles, 25 protocols of ongoing clinical trials were identified and included in the data synthesis of adherence reporting and assessment of the expected intensity of the multimodal intervention. These protocols were distributed geographically as follows: 8 from Europe, 7 from Asia, 5 from America, 4 from Australia, and 1 from Africa.

### Adherence definition in multimodal studies

Adherence is defined as the degree to which the person’s behavior corresponds with the agreed-upon recommendations from a healthcare provider [8]. It differs from compliance, which is the extent to which a patient’s behavior matches the prescriber’s advice, emphasizing obedience rather than actively choosing to make lifestyle changes. Adherence to multimodal interventions should encompass participation in intervention activities and lifestyle change, as both aspects impact cognitive change and are not directly interrelated [10]. Nonetheless, most studies thus far have focused solely on participation in intervention activities as a measure of adherence [9, 11–14]. Accordingly, good adherence has often been defined as completing at least 66% of prescribed interventions [9], a benchmark often used in behavioral interventions [17]. However, an arbitrary cutoff such as a simple percentage of 66% might not be informative of high adherence, as it depends on the overall amount of the intervention offered.

### Adherence reporting in multimodal studies

As shown in Table 1, there was significant heterogeneity in the reporting of adherence across the completed multimodal intervention studies. Adherence is commonly reported by intervention domain; however, certain studies, such as the Finnish Geriatric Intervention Study to Prevent Cognitive Impairment and Disability (FINGER) [9] and the GOIZ-ZAINDU [18], have also assessed simultaneous adherence to all assigned components, albeit with the use of cut-offs. This diversity in adherence reporting makes cross-trial comparisons of adherence to multimodal interventions difficult. This challenge could be improved by reporting average participation (mean (SD), in percentage units) to each intervention component.

**Table 1:** Reporting of adherence to multimodal interventions for preventing cognitive decline

Study (country; start-completion dates)	Multimodal intervention	Reporting of adherence to the multimodal intervention	Ref	
1. Completed studies with published results of adherence				
**FINGER** (Finland; 2009–2014)	Dietary counseling, exercise and cognitive training, and vascular risk factor monitoring	**Simultaneous adherence to all assigned intervention components (CV monitoring**,** Nutrition**,** Physical activity**,** Cognitive training)**: - 19.0% were adherent to at least 66% of prescribed treatment - 38.9% were adherent to at least 50% of prescribed program (i.e., *adherent*) - 42% were adherent to less than 50% of prescribed program (i.e., adherent to at least two components; *partially adherent*) - 21% were adherent to 0% of the prescribed program (i.e., *nonadherent*)	[9]	
		**CV monitoring visits**: - 94.6% were adherent to at least 50% of prescribed program - 92.9% were adherent to at least 66% of prescribed program **Nutrition**: - 90.0% were adherent to at least 50% of prescribed program **Physical activity**: - 60% were adherent to at least 50% of prescribed program **Cognitive training (individual + group sessions)**: - 47.2% were adherent to at least 50% of prescribed program - 24.7% were adherent to at least 66% of prescribed program	[9]	
		**Cognitive training (only individual sessions)**: - 63% participated in the cognitive training at least once - 20% completed at least 50% of the training sessions - 12% completed 100% of the training - Mean number of sessions (SD): 45.7 (54.95) - Median number of sessions: 15 (95%CI 6–23)	[62]	
**MAPT** (France; 2008–2014)	Integrated cognitive training, physical activity, and dietary advice and preventive consultations plus omega-3 PUFAs	**Multidomain sessions**: - 53.5% were adherent to at least 75% of prescribed program - 64.4% were adherent to at least 66% of prescribed program **Omega-3 capsules**: - 71.5% were adherent to at least 75% of prescribed program - 76.1% were adherent to at least 66% of prescribed program **Simultaneous adherence to all assigned intervention components (Multidomain sessions and Omega-3 capsules)**: - 50.7% were adherent to at least 75% of prescribed program - 61.1% were adherent to at least 66% of prescribed program **Cardiovascular consultations**: - 90.1% for the baseline visit - 71.9% for the 1-year visit - 62.3% for the 2-year visit **Multidomain dose**: - Range: 1–37 - Mean number of sessions (SD): 25.86 (9.26)	[9, 42]	
**eMIND** (France; 2017–2019)	Web-based multidomain lifestyle training intervention including cognitive training, exercise training, and nutritional advices	**Adherence to the MI**: Participants accessing all the three interventions (clicking on the multidomain contents in their personal agenda in the web-platform) for at least 75% of the requirements were considered adherent - 63.8% of participants adhered to the cognitive training - 60.3% of participants adhered to the nutrition intervention - 5.2% of participants adhered to the physical exercise intervention - 5.2% of participants adhered to all multidomain components - 53.4% of participants followed ≥ 50% of the requested frequency in exercise training (they connected once a week) - 75.8% of participants followed ≥ 50% of the requested frequency in cognitive training - 81% of participants followed ≥ 50% of the requested frequency in nutrition	[63]	
**preDIVA** (The Netherlands; 2006–2013)	Multidomain cardiovascular intervention (advice)	**Multidomain dose**: - 22.0% were nonadherent: Participants who on average had received less than 2 out of 3 annual intervention visits (< 66%) upon reaching a study endpoint (dementia/death/end of trial)	[14]	
**HATICE** (The Netherlands, Finland, France; 2015–2018)	Internet-based platform with remote support from a coach trained in motivational interviewing and lifestyle behavior advice	**Login frequency**: - 80% were adhered to the intervention (per-protocol analysis), defined as those who logged onto the platform in at least 12 out of 18 months study participation, and who set at least one goal or entered one or more measurements. - Reasonable uptake: Median of two logins per month in the platform with a wide range and a substantial proportion logging in more than five times a month), almost all participants setting at least one goal (with a considerable proportion up to three goals), and the majority of participants using the platform during the full study period. **Number of messages exchanged between coach and participant**: - 9.6% of the participants who completed the primary outcome sent 0 messages - 33.9% sent 1–5 messages - 29% sent 6–10 messages - 27.5% sent > 10 messages **Number of goals set**: - Median of 1 goal (IQR 1–2) - 9.8% set no goal - 47.3% set 1 goal - 34.0% set 2–3 goals - 8.9% set ≥ 4 goals	[26]	
**MIND-ADmini** (Sweden, France, Germany, Finland; 2017–2020)	Nutritional guidance, exercise, cognitive training, vascular/metabolic risk management and social stimulation Medical food	**Adherence to individual intervention components**: - 71.9% of participants adhered to the cognitive training intervention (attendance to 66% of group and individual sessions). - 68.8% of participants adhered to the nutrition intervention (attendance to 66% of sessions). - 81.3% of participants attended both the 3- and 6-month follow-up visits with the study nurse. - 78.1% of participants adhered to the exercise intervention (attendance to 40% of group-based gym sessions). - 87.1% of participants consumed ≥ 60% of medical food product (measured through a study product diary). - 78.1% and 87.1% of participants in the lifestyle and lifestyle + medical food arms were overall adherent, respectively. **Overall adherence to the intervention (composite measure of participation in different intervention components)**: - 78.1% of participants in the lifestyle intervention arm attended a minimum of 40% of sessions per domain, in at least 2/4 domains (exercise, nutrition, cognitive training and monitoring of vascular/metabolic risk factors). - 87.1% of participants in the lifestyle + medical food intervention arm attended a minimum of 40% of sessions per domain, in at least 2/4 domains, and additionally consumed at least 60% of the medical food study product. **Overall adherence to healthy lifestyle changes**: composite healthy lifestyle score (ranges 0–8 points) based on the FINGER study(64). A score from 0 to 2 was assigned to each tertile of the healthy dietary intake score, physical activity level, cognitive and social activities, and cardiovascular risk burden	[64, 65]	
**AgeWell.de** (Germany; 2018–2022)	Nutritional counseling, enhancement of physical and social activity, cognitive training, and the management of cardiovascular risk factors (overweight, smoking).	**Adherence to the MI**: it was evaluated by asking to what extent participants were able to reach their goals in the domains of nutrition, physical activity, cognitive activity, and social activity (response options: not at all [0]–absolutely [4]). Adherence to all intervention components was summed across all seven points in time, leading to a score ranging from 0 to 28. **Nutrition component (0–4 points)**: - Mean adherence (SD): 2.80 (0.71) **Cognitive activity (0–4 points)**: - Mean adherence (SD): 2.90 (0.78) **Physical activity (0–4 points)**: - Mean adherence (SD): 2.66 (0.82) **Social activity (0–4 points)**: - Mean adherence (SD): 2.81 (0.76) **All components (0–28 points)**: - Not reported	[25]	
**GOIZ ZAINDU** (Spain; 2018–2020)	Nutrition counseling, management of vascular risk factors, physical activity, and individual- and group-based cognitive intervention	**Overall adherence to the MI**: - 54.7% of participants had high adherence (attendance to all intervention components was higher than 50%) - 17.2% had partial adherence (attendance to at least 30% of activities of all intervention components) - 18.8% had low adherence (attendance to any intervention components was lower than 30%) - 9.4% had very low adherence (attendance to less than 30% to two or more intervention components) **Nutrition component**: - 73.4% completed at least 2/3 nutrition counseling visits - Mean adherence: 74.5% **CV monitoring component**: - 67.2% completed at least 2/3 cardiovascular monitoring visits - Mean adherence: 73.44% **Individual cognitive training**: - 64.1% completed > 50% of the cognitive training individual materials - Mean adherence: 55.47% **Group cognitive training**: - 70.0% attended > 50% of cognition stimulation workshops - Mean adherence: 54.78% **Physical exercise**: - > 75% of participants reported practicing physical exercise at least twice a week during the intervention period. Mean adherence: 76.56%	[18]	
**ASPIS** (Austria; 2010–2014)	Intensive control and motivation for better compliance with prescribed evidence-based medication, regular blood pressure measurements, healthy diet, regular physical activity and cognitive training.	**Dietary individual counseling visits (7 in total)**: - Median (IQR) attendance: 5 (3–5) **Dietary group counseling visits (7 in total)**: - Median (IQR) attendance: 7 (3–7) **Physical activity group meetings (9 in total)**: - Median (IQR) attendance: 8 (4–11) **Cognitive group meetings (24 in total)**: - Median (IQR) attendance: 12 visits (5.5–17)	[66, 67]	
**StayFitLonger** (Switzerland, Canada, Belgium; 2019–2021)	Computerized physical and cognitive training exercises, plus social interactions through access to a moderated Chat Room, psychoeducational content, and gamification elements.	**Mean time weekly spent using the program**: - 2.6 (SD = 0.3) hours	[68]	
**SMARRT** (USA; 2018–2022)	Multidomain health coaching sessions offered every 4 to 6 weeks	**Health contacts**: - Mean (SD): 18.8 (5.5) - Range: 1–28	[69]	
**COCOA** (USA; 2018–2022)	Remotely coached multimodal lifestyle intervention	**Adherence to the MI**: - 86% of participants remained actively engaged with their coach longer than one year - 20.7% of participants remained actively engaged with their coach after two years	[70]	
**Meng X**,** et al. 2024** (China; 2017–2017)	Online education, cognitive training and community activities	**Adherence to online material** (number of times that the participants completed the readings): - Mean adherence: 70% **Adherence to computer-based cognitive training** (number of times that the participants completed the trainings): - Mean adherence: 26.1% **Adherence to face-to-face activities** (% of intervention activities attended): - Mean adherence: 65.2%	[71]	
**J-MINT** (Japan; 2019–2022)	Multicomponent intervention including physical exercise, nutrition counseling and cognitive training	**Physical activity** - Mean adherence: 83% (64.9 ± 15.8 out of 78 sessions) - 84% of participants adhered to ≥ 70% of group-based physical exercise sessions **Cognitive training** - Mean adherence: 44.4% (69.2 ± 96.9 out of 156 sessions) - 18% of participants adhered to 100% of the cognitive training sessions	[35, 72]	
**Bae S**,** et al. 2019** (Japan; 2017–2017)	Multicomponent intervention including physical, cognitive, or social activity sessions	**Adherence to the MI**: - 70.2% of participants attended to the MI	[73]	
**SUPERBRAIN** (South Korea; 2019–2020)	Vascular risk monitoring and management, cognitive training and social activity, physical exercise, nutritional guidance, and motivational enhancement	- **Adherence to the MI**: 94.5% (95%CI 91.4, 97.6%) - **Vascular and metabolic program**: 98.0% - **Cognitive training**: 97.4% - **Social activity**: 95.9% - **Physical exercise**: 91.0% - **Nutrition**: 94.2% - **Motivational enhancement program**: 97.7%	[74]	
**SINGER-Pilot** (Singapore; 2018–2020)	Dietary counseling, exercise and cognitive training, and vascular risk factor monitoring	**CV monitoring visits**: - 100% of participants completed 100% of the sessions **Home-based exercise**: - 53% of participants completed > 50% of the sessions **Food diary completion**: - 97% of participants filled the food diary in > 50% of their meals for the three required days at each time point **Cognitive training**: - 81% of participants completed > 50% of the training	[75]	
**Ng PEM**,** et al. 2021** (Singapore; 2018–2020)	Computerized cognitive training, physical-cognitive dual-task exercises (in small group activities) and nutritional guidance (through mobile app)	**Physical-cognitive dual task sessions**: - Mean adherence (SD): 75.78% (19.13) **Small group activities**: - Mean adherence (SD): 78.90% (20.25) **Computerized cognitive training**: - Mean adherence (SD): 70.99% (23.77) **Nutritional mobile application**: Used by 15% of participants	[76]	
**S-FIT** (Singapore; 2009–2014)	Cognitive training, physical training, and nutritional intervention (dietary supplement)	**Adherence to the MI**: measured monthly by estimating the proportion of dietary supplements consumed and training sessions completed. - Mean adherence: 88%	[77]	
***Ongoing studies within the WW-FINGERS network with prespecified reporting of adherence in the study protocol***				
**LETHE** (Austria, Finland, Italy, Sweden)	Nutritional counseling, exercise, cognitive training, management of vascular/metabolic risk factors, social activity, and sleep and relaxation	**Individual components**: - Usage of and engagement with the mobile app and smartwatch (frequency and duration of logins). o During the first 6 months, 50.5% of participants in the intervention group used the full app daily. The median duration of a single session of app usage was 42.1 s in the intervention group. Most participants (98.7%) completed a digital cognitive testing one month after baseline visit. - Participation in study visits and intervention-related activities and meetings. **Overall adherence to the intervention**: - Adherence to a healthy lifestyle: composite score developed in FINGER, based on self-reported data on exercise, diet, smoking and alcohol, and social and cognitive activity.	[31, 78]	
**PENSA** (Spain)	Nutritional counseling, physical activity, cognitive training, psychoeducation sessions and social stimulation sessions.	**Nutrition counseling visits**: - Mean percentage of sessions (SD): 90.3% (29.6) - 34.4% were adherent to 100% of sessions - 80.2% were adherent to 89% of sessions (8 out of 9 visits) - 99% were adherent to 78% of sessions (7 out of 9 visits) **Gymnasium classes**: - Mean percentage of sessions (SD): 62.1% (38.2) - 45.8% were adherent to ≥ 75% of sessions - 70.0% were adherent to ≥ 50% of sessions **Cognitive training**: - Mean percentage of sessions (SD): 72.6% (30.8) - 60.4% were adherent to ≥ 75% of sessions - 83.3% were adherent to ≥ 50% of sessions **Psychoeducation sessions**: - Mean percentage of sessions (SD): 79.0% (40.8) - 75.0% were adherent to ≥ 70% of sessions **Ecological Momentary Assessments (EMAs)**: average weekly adherence, calculated as the number of days that participants completed the dietary EMAs questionnaire divided by 7 days - Mean weekly compliance (SD): 89.0% (17.5) - 93.4% had ≥ 75% of compliance **Fitbit**: number of complete observations, defined as at least 600 min of valid minute heart rate signal per day (i.e., wear time indicator) - Mean monthly valid data (SD): 84.1% (36.6) - 60.4% had valid data in ≥ 90% of days - 79.2% had valid data in ≥ 75% of days - 92.7% had valid data in ≥ 50% of days **Epigallocatechin-3-gallate (EGCG)**: presence of ≥ 10 ng/mL of EGCG in plasma - 93.5% of compliance at 6 months - 85.1% of compliance at 12 months	[32] *(unpublished data)*	
**MET-FINGER** (UK, Finland, Sweden)	1. Dietary counseling, exercise and cognitive training, and vascular and metabolic risk factor monitoring 2. Metformin when appropriate	**Individual components**: - **Cognitive training**: attendance at the cognitive training sessions including automatic recordings of computer program use, and group sessions attendance. - **Nutrition**: attendance at the 7 group and 3 individual nutrition sessions. - **Vascular and metabolic risk factors monitoring**: attendance to 6 individual consultations (at least 3 with a study physician). - **Physical exercise**: participation in group exercise sessions. - **Metformin**: adherence to target dose and compliance to treatment. **Overall adherence to the intervention**: calculated through composite measure of participation in different intervention components.	[36]	
**LatAM-FINGERS** (Argentina, Brazil, Bolivia, Chile, Colombia, Costa Rica, Cuba, Dominican Republic, Ecuador, Mexico, Paraguay, Peru, Puerto Rico, and Uruguay)	Exercise, diet, cognitive/social stimulation, and cardiovascular health	**Implementation: level of fidelity to the intervention protocol estimated on the basis of**: - % attendance to team meetings - % physical activity sessions completed - % cognitive training sessions completed - % telephone contacts completed - % health monitoring visits completed - Participant’s self-reported adherence to the intervention	[30]	
**Africa-FINGERS** (Kenya and Nigeria)	Exercise, diet, cognitive/social stimulation, and cardiovascular health	Intervention compliance/adherence will be measured via attendance records at group intervention meetings (e.g., for physical, social, and cognitive activities), questionnaires on dietary changes/intake, subjective reports of satisfaction with progress to monitor skills/brain training, and regular vascular and metabolic risk monitoring (blood pressure, body mass index, and blood work).	[33]	

*The studies are sorted by country and continent of origin*

*CV = cardiovascular. IQR = interquartile range. MI = multimodal intervention. PUFA = polyunsaturated fatty acids*

On the other hand, ensuring consistency in reporting lifestyle changes across multimodal interventions can be challenging due to the considerable heterogeneity of the assessment tools used to measure lifestyle changes. A solution to this harmonization challenge could be the use of dementia risk scores such as The Cardiovascular Risk Factors, Aging, and Incidence of Dementia (CAIDE) risk score or the Lifestyle for Brain Health (LIBRA) index [19]. These scores can be calculated uniformly across studies irrespective of the various measurement instruments used to evaluate lifestyle or cardiovascular risk factors [20, 21]. Moreover, they have been suggested as surrogate outcomes for lifestyle-based multimodal prevention trials because they may register changes in dementia risk before detectable cognitive changes [22, 23]. However, risk scores often attribute points using categorical scoring systems, which might reduce responsiveness, as large changes in individual risk factors may not be registered if these changes do not cross the categorical cutoff points [22]. To improve their performance, it has been proposed to use continuous scoring systems (crude and weighted z-score versions), taking into account all changes in risk factors, not only those crossing specific cutoff values [22]. Using this approach, the LIBRA index demonstrated greater responsiveness to change, than did the CAIDE dementia risk score, as it includes a larger number of modifiable risk factors and a broader range of scores [22]. However, risk scores often put very limited weight on the lifestyle changes most frequently targeted in multimodal interventions (e.g., CAIDE has physical activity only, and LIBRA has physical activity, cognitive activity and diet) and contain many factors that cannot be changed (the effect of multimodal interventions on cardiovascular risk factors is modest, particularly when medications are not a part of the program).

### Intensity of multimodal interventions

A measure of the intensity of the intervention that combines the dose delivered (i.e., total number of sessions) and the length of the intervention may be more useful for comparing adherence to different multimodal interventions. Among large multimodal intervention studies, the FINGER is the only one that demonstrated benefits on cognition [24]. The Multimodal Alzheimer Preventive Trial (MAPT) [9] and the Prevention of Dementia by Intensive Vascular Care (PreDIVA) [14], for example, reported no intervention effect on their primary cognitive outcomes. In addition to differences in the target population (at-risk individuals in the FINGER, frail individuals with subjective cognitive impairment in the MAPT, and the general population in the PreDIVA), these three studies differed substantially in terms of intervention intensity and delivery mode (structured intervention programs in the FINGER and MAPT, and mainly self-guided intervention in the PreDIVA). The expected intensity of the FINGER study, calculated as the ratio between the prescribed dose (number of prespecified sessions) and the length (months), was 10.6 points. In contrast, the MAPT had an intensity of 1.2 points, and the PreDIVA had an even lower intensity of 0.3 points (Table 2).

**Table 2:** Intensity of multimodal intervention studies

Study (country; start-completion dates)	MI population	Length (mo)	Multimodal Dose^1^ (number of visits or sessions)	Expected intensity (Dose _expected_/ Length)	Observed intensity (Dose _observed_/ Length)	Main delivery mode	Cognitive outcome results	Ref
Completed studies								
**FINGER** (Finland; 2009–2014)	*N* = 631 (60–77 years, CAIDE dementia risk score ≥ 6, and cognitive performance at the mean level or slightly lower than expected for age)	24	**Expected: 256 sessions in total** - 150 Cognitive training (6 group sessions and 144 training exercises) - 88 Physical exercise sessions (80 individual gym training sessions and 8 individual muscle training sessions) - 9 Nutrition counseling visits (3 individual and 6 group sessions) - 9 CV consultations (2 doctor and 7 nurse consultations)	10.6		Structured program	Intervention had beneficial effect on the NTB composite	[9, 10, 24, 62]
**MAPT** (France; 2008–2014)	*N* = 837 (≥ 70 years, MMSE ≥ 24, with memory complaints, limitation in one IADL or slow gait speed)	36	**Expected: 43 sessions in total** (38 group sessions + 5 individual interviews) - 14 Multidomain sessions (combined cognitive training, nutrition and physical activity advice) - 14 Cognitive training sessions - 6 Physical activity advice - 3 Nutritional advice - 6 General health advice/Cardiovascular consultations - Omega-3 capsules (2 capsules/day) **Observed: 28.86** (mean number of sessions)	1.2	0.8	Structured program	No effect of intervention on global cognition measured with a composite Z score combining four tests. No effect of intervention on other secondary cognitive outcomes.	[9, 42]
**eMIND** (France; 2017–2019)	*N* = 60 (≥ 65 years, MMSE ≥ 24, with subjective memory complaints)	6	**Expected: 117 sessions** Participants were requested to follow both exercise and cognitive training twice a week, and nutritional advices every fifteen days;	19.5		Self-guided	No differences on global cognition measured with a composite Z score combining four tests. No effect of intervention on other cognitive outcomes.	[63]
**preDIVA** (The Netherlands; 2006–2013)	*N* = 1293 (70–78 years without dementia)	72	**Expected: 18 visits in total** (every 4 months) with practice nurse and general practice where participants were assessed for CV risk factors and blood glucose and lipid concentrations (every two years). On the basis of these assessments, participants received individually tailored lifestyle advice.	0.3		Self-guided	No effect of intervention on dementia incidence.	[14, 61]
**HATICE** (The Netherlands, Finland, France; 2015–2018)	*N* = 1194 (≥ 55 years without dementia, with CV risk factors, history of CVD or diabetes)	18	**Expected: 72 logins in total** (4 logins per month were expected) **Observed: 32 logins in total** (median of 1.8 logins per month)	4.0	1.8	Self-guided	No differences between groups in the MMSE or a composite Z score of seven cognitive tests. No effect of the intervention on any of the individual cognitive tests.	[26, 43]
**MIND-ADmini** (Sweden, France, Germany, Finland; 2017–2020)	*N* = 63 (60–85 years, prodromal AD)	6	**Expected: 106 sessions** - 6 nutrition counseling visits (3–4 group and 3 individual sessions) - 48 exercise sessions (2 times/week) - 50 cognitive training sessions (2–3 group sessions and 48 individual training sessions twice a week) - 2 CV risk consultation visits at 3 and 6 months **Medical food** (a 125 ml once-a-day milk-based drink)	17.7		Structured program	Not reported	[65]
**AgeWell.de** (Germany; 2018–2022)	*N* = 546 (60–77 years, CAIDE ≥ 9, without dementia)	24	**Expected: 527 in total** - 208 Physical activity sessions (exercises for strength/flexibility to be conducted at home twice a week) - 312 Cognitive training sessions (15 min, 3 times per week) - 7 Nutrition counseling visits (2 face-to-face and 5 via telephone) led by study nurses (0, 2, 4, 8, 16 and 20 months) - Management of vascular risk factors and individual goals for social activities in the regular general practitioner (GP) visits	22.0		Self-guided	No effect of intervention on global cognitive performance measured with a composite Z score combining five tests. In sensitivity analyses, beneficial intervention effects were observed on the domain of social cognition.	[25]
**GOIZ ZAINDU** (Spain; 2018–2020)	*N* = 64 (+ 60 years, CAIDE ≥ 6, no dementia, cognitive performance below-than-expected or with MCI)	12	**Expected: 156 sessions** - 3 cardiovascular risk monitoring visits (month 3, 6, 9) - 5 nutritional visits (month 1, 3, 6, 7, 9) - 13 group-based cognitive stimulation sessions - 135 individual cognitive training sessions (3 times/week for 10 months) - Recommendations to practice 2 to 6 times/week of physical exercise) **Observed: 99 sessions** - 2 cardiovascular visits (3 with 67.2% of average participation) - 3.7 nutritional counseling visits (5 with 73.4% of adherence) - 7.1 group-based cognitive stimulation sessions (13 with 54.8% of adherence) - 86.5 individual cognitive training sessions (135 with 64.1% of adherence) *Self-reported adherence	13	8.3	Structured program	No effect of intervention on global cognition measured with the modified NTB composite (mixed models of repeated measures analyses). However, the intervention reduced the risk of cognitive decline for the NTB executive function score and the NTB processing speed score.	[18]
**ASPIS** (Austria; 2010–2014)	*N* = 101 (40–80 years, MMSE ≥ 24, within 3 months after an acute stroke)	24	**Expected: 45 sessions** - 13 dietary counseling visits (7 group and 6 individual visits) - 8 physical exercise group meetings. - 24 cognitive training group sessions (monthly) **Observed: 32 sessions** - 12 dietary counseling visits (5 individual and 7 group; median adherence) - 8 physical activity group meetings (median adherence) - 12 cognitive group meetings (median adherence)	1.9	1.3	Structured program	No effect of intervention on global cognition measured with the ADAS-cog. No effect of intervention on cognitive decline or other secondary cognitive outcome variables.	[66, 67]
**StayFitLonger** (Switzerland, Canada, Belgium; 2019–2021)	*N* = 59 (≥ 60 years, MoCA ≥ 26, and Fried’s frailty index < 3)	6	**Expected: 156 sessions in total** During the 26 weeks of intervention, participants were asked to engage in physical exercise 3 days per week for 30–45 min and cognitive exercise for at least three 15-min sessions per week, and through a chat room had opportunities for social and contributing interactions, and psychoeducation content	26.0		Self-guided	In the overall sample, no effect of intervention on global cognition measured with the ZAVEN composite score. No effect of intervention on executive function, processing speed, or memory composites.	[68]
**SMARRT** (USA; 2018–2022)	*N* = 82 (70–89 years and with ≥ 2 of 8 targeted risk factors)	24	**Expected: 28 health coaching contacts** (maximum number of contacts) - Health coaching sessions offered every 4 weeks during the first 3 months, and every 6 weeks for the final 15 months **Observed: 18.5** (mean number of contacts)	1.2	0.8	Self-guided	Intervention had beneficial effect on the modified NTB composite	[69, 79]
**COCOA** (USA; 2018–2022)	*N* = 31 (≥ 50 years with early AD)	24	**Expected: 778 sessions**: - 24 calls by a dietitian or nurse. - 24 text or email communications - 730 sessions of cognitive training (30 min/day during the 24 months)	32.4		Self-guided	Intervention had beneficial effect on the Memory Performance Index	[70, 80, 81]
**COMBAT** (China; 2019–2021)	*N* = 86 (≥ 60 years at-risk)	9	**Expected: 39 sessions in total** - Multidomain training program consisting of mindfulness meditation, cognitive training, physical exercise, and nutrition counseling for 9 months, delivered with weekly group sessions at the community hospitals and self-monitoring homework.	4.3		Structured program	Intervention had beneficial effect on global cognition measured with a composite Z score combining seven tests	[82]
**Yang Q-h**,** et al. 2022** (China; 2019–2021)	*N* = 61 (≥ 65 years with MCI)	6	**Expected: 102 sessions** - 6 individual nutrition counseling visits. - 44 physical activity sessions (facility based; once/week for the first month and twice/week for months 2–6). - 48 cognitive training sessions (twice/week). - 4 vascular risk monitoring visits.	17.0		Structured program	Intervention had beneficial effect on the MoCA total score	[83, 84]
**Meng X**,** et al. 2024** (China; 2017–2017)	*N* = 48 (≥ 60 years at risk)	6	**Expected: 169 sessions** - 153 online educational program, including 27 articles and 126 messages. - 12 cognitive training sessions (1/week, for 12 weeks). - 1 group discussion on dementia-related themes - 3 outdoor aerobic activities - 3 one-to-one voluntary services (only for 11 participants; not considered in the total dose) **Observed: 115 sessions** - 109 online educational program (70% adherence) - 3 cognitive training sessions (26% adherence) - 1 group discussion (74% adherence) - 2 outdoor aerobic activities (65% adherence)	28.2	19.2	Self-guided	Intervention had beneficial effect on global cognition measured with a composite Z score combining four tests (PACC)	[71]
**J-MINT** (Japan; 2019–2022)	*N* = 265 (65–85 years with MCI)	18	**Expected: 245 sessions** - 78 physical exercise sessions in group - 11 nutrition counseling visits (8 telephone and 3 in-person meetings) - 156 cognitive training sessions (9 months of cognitive training at least 4 times/week) **Observed: 146 sessions** - 64.9 ± 15.8 physical exercise sessions (83% of adherence) - 69.9 ± 96.8 cognitive training sessions - No information on adherence rates to nutrition (11 sessions planned)	13.6	8.1	Structured program	No differences in cognitive performance measured with a composite Z score combining seven tests. Benefits on executive function/processing speed	[35, 72]
**J-MIND-Diabetes** (Japan; 2019–2022)	*N* = 81 (70–85 years with type 2 diabetes and with MCI to mild dementia)	18	**Expected: 48 sessions** - 39 group-based physical exercise sessions - 9 individual nutritional counseling sessions (once every two months) - Recommendations to go out at least 3 times/week to promote social participation - Personalized goals for management of diabetes	2.7		Structured program	No differences in cognitive performance measured with a composite Z score combining eight tests	[85]
**Bae S**,** et al. 2019** (Japan; 2017–2017)	*N* = 41 (≥ 60 years with MCI)	6	**Expected: 48 sessions** - Integrated 90-minute multimodal sessions twice weekly for 24 weeks (16 physical activity sessions, 16 cognitive activities sessions, and 16 social activities sessions)	8.0		Structured program	No differences in cognitive performance measured with the MMSE. However, the intervention had beneficial effect on spatial working memory.	[73]
**SUPERBRAIN** (South Korea; 2019–2020)	*N* = 51 (older adults with memory complaints)	6	**Expected: 72 sessions in total** - Participants visited a study facility three times a week to perform all intervention programs in group or individual sessions (during 24 weeks) **Observed: 68 sessions** (72 sessions with average adherence of 94.5%)	12	11.3	Structured program	Intervention had beneficial effect on global cognition measured with the RBANS total scale index score.	[74]
**Park JE**,** et al. 2019** (South Korea; 2016–2017)	*N* = 13 (≥ 60 years, pre-frail/frail)	6	**Expected: 13 sessions** - 8 MI sessions: 4-week group-based intensive program - 5 Monthly monitoring sessions (20 weeks) during the maintenance program	2.1		Structured program	No differences in global cognitive performance measured with the CERAD-TS battery.	[86]
**Ng PEM**,** et al. 2021** (Singapore; 2018–2020)	*N* = 96 (≥ 55 years at risk of cognitive impairment)	6	**Expected: 48 sessions in total** (biweekly sessions) - Cognitive training and physical activity: 31% physical-cognitive dual-task exercises and 69% cognitive sessions, of which 19% were based on small group activities and 50% were computerized cognitive training. Physical training of moderate intensity was conducted in supervised groups of 6 to 10 participants - Nutritional guidance was intended to be on-going via a mobile application throughout the length of the intervention, with one a month face-to-face nutritional talk with a dietitian	8.0		Self-guided	No effect of intervention on global cognition measured with the RBANS total score. No effect of intervention on specific cognitive domains.	[76]
**S-FIT** (Singapore; 2009–2014)	*N* = 49 (≥ 65 years, frailty, MMSE > 23)	6	**Expected: 48 in total** - 24 cognitive training sessions: 2-hour duration weekly group training sessions - 24 physical exercise: Moderate intensity physical exercise on 2 days per week in supervised groups for 12 weeks, followed by 12 weeks of home-based exercises - Daily nutritional supplement **Observed: 42** (72 sessions with 88% of adherence)	8	7	Structured program	No effect of intervention on global cognition measured with the RBANS total scale index score. Intervention had beneficial effect on visuospatial construction.	[77]
**SINGER-Pilot** (Singapore; 2018–2020)	*N* = 34 (≥ 65 years with mild-to-moderate frailty)	6	**Expected: 81 sessions** - 4 dietary intervention sessions (2 group, 2 individual) - 3 vascular monitoring visits - 74 cognitive training sessions (72 cognitive training sessions plus 2 memory group talks) - Home-based physical exercise recommendations (twice weekly)	13.5		Structured program	Not reported	[75]
**THISCE** (Taiwan; 2014–2016)	*N* = 549 (≥ 65 years, at risk)	12	**Expected: 16 sessions** - 16 Multidomain sessions: 4 during the first month, 2 during the second month, and 1 month during months 3–12	1.3		Structured program	No differences in cognitive performance measured with the MoCA test.	[87]
***Ongoing studies within the WW-FINGERS network***								
**PENSA Study** (Spain)	*N* = 104 (60–80 years, *APOE*-ɛ4 carriers with subjective cognitive decline)	12	**Expected: 245 sessions in total** - 144 individual cognitive training sessions (12/month) - 9 individual nutrition counseling visits - 72 group exercise sessions - 10 psychoeducation group sessions - 10 social and cognitive stimulation activities - **EGCG intake** (1–2 times/day) - **Ecological momentary assessments-EMAs** (daily) - **Activity tracker** (daily) **Observed**: **175 sessions in total** - 104 cognitive training sessions (144 with 72.6% average adherence) - 8.1 individual nutrition counseling visits (9 visits with 90.3% of adherence) - 44.7 exercise sessions (72 sessions with 62.1% average adherence) - 7.9 psychoeducation group sessions (10 sessions with 79% of adherence) - 10 social stimulation sessions (no data on compliance)	20.4	14.5	Structured program		[32]
**CITA GO-ON** (Spain)	*N* = 547 (60–85 years, CAIDE ≥ 6, nondemented but with low performance in at least one of 3 cognitive tests)	24	**Expected: 340** - 6 cardiovascular risk monitoring visits (1 visit every 4 months) - 6 group-based nutrition counseling visits - Recommendations to perform physical activity at home (30 min/day) - 16 group-based cognitive training sessions - 312 individual cognitive training sessions (15–20 min, 3 days/week)	14.1		Structured program		ClinicalTrials.gov Identifier: NCT04840030 [34]
**FINGER-NL** (The Netherlands)	*N* = 603 (60–80 years at risk)	24	**Expected: 112 sessions** The lifestyle intervention consists of 7 lifestyle domains including physical exercise, cognitive training, management of metabolic and vascular risk factors, nutritional counseling, sleep counseling, stress management, and social activities; and Souvenaid^®^. - 21 physical activity sessions (9 online group meetings, 7 study-site meetings and 5 personal lifestyle coach sessions). - 21 cognitive training sessions (5 online group meetings, 2 personal lifestyle coach sessions, and 6 individual sessions provided via the digital intervention platform; plus 2 booster sessions during the second year, each including 2 online group meetings and 2 individual sessions). - 11 CV risk management visits (2 online group meeting, 4 at study-site group meetings and 6 personal lifestyle coach sessions) - 34 nutritional counselling visits (5 online group meeting, 1 at study-site group meeting, 24 individual online sessions and 4 personal lifestyle coach sessions) - 5 sleep counselling visits (1 online group meeting, 1 personal lifestyle coach sessions, 2 individual sessions provided via the digital platform; plus 1 booster session that includes an online group meeting) - 12 stress-management visits (7 study-site group meetings, 2 online group meetings and 3 individual online sessions) - 8 social activities (group meetings)	4.7		Mixed		[39, 40]
**MET-FINGER** (UK, Finland, Sweden)	*N* = 300 (60–79 years, *APOE* ε4-enriched population at increased risk of dementia)	24	**Expected: 310 sessions** - 10 dietary sessions (7 group and 3 individual sessions) - 144 physical activity sessions (92 in a gymnasium and 52 online) - 150 cognitive training sessions (6 in group and 144 independent training exercises) - 6 CV monitoring sessions (3 practical and 3 medical consultation) **Metformin** (2 times/day)	12.9		Structured program		[36]
**LETHE** (Austria, Finland, Italy, Sweden)	*N* = 78 (60–77 years with increased dementia risk and sufficient digital readiness)	24	**Expected: 532 sessions (approx.)** - 5 dietary sessions (3–4 group and 1–2 individual sessions) - 209 physical activity sessions (1 in-person group session plus 1 session/week of independent strength training, and 1–5 sessions/week of independent aerobic training) - 315 cognitive training sessions (3 in group and 312 independent training) - 2–4 CV monitoring sessions	22.2		Mixed		[31]
**BRAIN-DIABETES** (Ireland)	*N* = 35 (50 + years with type 2 diabetes)	6	**Expected: Not available** - 4-month active lifestyle program in the community followed by 2 months of engaging in a self-directed lifestyle program: o 5 diet sessions:1 online 90-min session with a nutritionist plus 4 online/telephone review meetings o 66 cognitive training sessions (4 individual cognitive training sessions per week for 30 min plus 2 introductory training sessions) o Remotely administered exercise intervention involving 1 online session with an instructor plus personalized recommendations to perform exercises at home that will increase in frequency and intensity over the first 4 weeks (number of sessions not specified) o 3 CV monitoring sessions			Mixed		ClinicalTrials.gov Identifier: NCT05304975 [88]
**PDP** (Luxembourg)	*N* = 450 (31–90 years; implementation study targeting individuals with SCD or MCI).		**Expected dose: Not available** - Voucher system to provide access to the program activities, with a broad range of choices for cognitive training, physical activity, and social activities, individual sessions with a dietician, and counselling sessions with a psychologist.			Structured program		[89]
**U.S. POINTER** (USA)	*N* = 1000 (at-risk older adults)	24	**Expected: 769 sessions** - 38 weekly meetings: The structured (STR) program included weekly meetings for the first 4 months, then 2x/month for 2 months, and then monthly, with specific goals for physical activity, diet, cognitive/social challenge and health monitoring. Meetings were performed in groups of 12–15 participants. - 416 physical activity sessions: 4 times/week of physical activity (4 times/week of moderate-to-high intensity aerobic exercise for 30 min, 2 times/week of resistance training with weight for 15–20 min, and 2 times/week of stretching/balancing activities) - 312 Computer-based cognitive training (3 times/week) and home-based cognitively and socially challenging activities (via Team Meetings) - 3 Health coaching visits (participants meet with a study Medical Advisor every 6 months)	32.0		Structured program		[27, 28]
**Can Thumbs Up** (Canada)	*N* = 350 (60–85 years, cognitively unimpaired or MCI with at increased risk of dementia)	12	**Expected: Not available** Fully remote web-based educational intervention (Brain Health Support Program intervention for 45 weeks). The program content is provided progressively to deliver new weekly content of 40 min. Participants are invited to go over the material at their own rate over the week.			Self-guided		[90]
**LatAM-FINGERS** (Argentina, Brazil, Bolivia, Chile, Colombia, Costa Rica, Cuba, Dominican Republic, Ecuador, Mexico, Paraguay, Peru, Puerto Rico, and Uruguay)	*N* = 600 (60–79 years at risk for dementia)	12	**Expected: 364 sessions** - 148 physical activity sessions (4 educational meetings + 144 group exercises on a regular basis; flexible protocol including 2–4 times/week sessions**)** - 4 dietary sessions (4 educational meetings + diet with nutritionist follow-up) - 208 cognitive training sessions (4 weekly sessions) - 4 medical monitoring sessions (4 educational meetings plus individual medical appointments)	30.3		Structured program		[30]
**Africa-FINGERS** (Kenya and Nigeria)	*N* = 300 (50–85 years at risk for dementia)	24	**Expected: 359 sessions** - 10 nutritional counselling visits (3 individual and 7 group sessions) - 312 physical activity sessions (2–4 sessions/week) - 32 cognitive training sessions (8 group sessions and 24 individual sessions performed once/week for ~ 6 months) - 5 individual cardiovascular monitoring sessions	15.0		Structured program		[33]
**SINGER** (Singapore)	*N* = 600 (60–77 years, at risk)	24	**Expected: 254 sessions** - 9 diet counseling sessions: 6 group-based nutrition advocacy workshops over 12 months plus 3 individual-based nutrition training sessions - 88 physical exercise sessions: once to twice weekly physical activity sessions (modified FINGER exercise program) - 154 cognitive training sessions: 10 cognitive training workshops plus 144 computer-based cognitive training sessions - 3 vascular risk factors management sessions	10.5		Structured program		[37]
**MYB-Maintain Your Brain** (Australia)	*N* = 4250 (55–77 years with risk factors for dementia)	36	**Expected: Not available** Online: 4 modules (physical activity, nutrition, peace of mind, and brain training) administered based on individual risk profiles. Participants will complete their assigned modules sequentially, noting that the total number of modules varies depending on the respective individual’s risk factors. In practice, this will translate to a minimum of two modules and a maximum of four modules. Each module lasts 10 weeks. Upon completing a module, booster sessions (specific to each module) and ongoing monitoring will then continue for up to three years.			Self-guided		[91]
**AU-ARROW** (Australia)	*N* = 300 (55–79 years with risk factors for cognitive decline and dementia)	24	**Expected: 764 sessions** - 16 group education meetings during the first month. - 416 physical exercise sessions in a gymnasium: four times/week (with at least 2 group classes per week). - 23 diet counselling sessions via the group education meetings plus monthly individual follow-up consultations with a dietitian starting at month 2 (phone calls). - 264 computer-based cognitive training sessions. Brain training exercises start at month 3 and are performed at home four times/week. - 3 medical monitoring consultations (every 6 months) - 23 group meetings (1 session/month, starting at month 2) - 19 monthly group meetings to provide further health education and support starting at week 6.	31.8		Structured program		[29]
**My-AGELESS** (Malaysia)	*N* = 165 (60–80 years with cognitive frailty)	24	**Expected: not available** - 12 Nutritional guidance sessions: 3 individual and 9 group sessions - Psychosocial interventions (number of sessions not specified) - Physical training sessions: progressively increasing from one to five times a week over a period of 21 weeks - 260 cognitive training sessions (3 times a week for 20 months)			Structured program		[92]
**FINOMAIN** (Philippines)	*N* = 300 (≥ 60 years with type 2 diabetes)	12	**Expected: 110 sessions** - 104 physical/cognitive exercise: dance called INDAK (Improving Neurocognition through Dance and Kinesthetics): 1 h, twice per week - 3 Nutrition counseling visits (every 3 months) - 3 Vascular risk management (every 3 months)	9.2		Structured program		[38]
**MIND-CHINA** (China)	*N* = 760 (60–79 years)	NA	- Vascular care (blood pressure and fasting blood glucose are monitored every 2 months, blood lipids are monitored every 12 months, and group health education is held once every 6 months) - Lifestyle and dietary guidance to improve treatment and control of hypertension, diabetes, and dyslipidemia - Group walking activities (five times a week with each exercise lasting for 20–30 min) - Personalized leisure activities (paper cutting and chess games) - Cognitive training (three times a week, 20 min each time, each time includes 5 games, 4 min/game, and the training is carried out for 6 months each year, a total of 72 times)					[93] Chinese Clinical Trial Registry number ChiCTR1800017758
***Ongoing studies outside the WW-FINGERS network***								
**MINE** (China)	*N* = 360 (≥ 50 years, MoCA < 26 points and MMSE > 22 points)	6	**Expected: 176 sessions** - 72 Exercise training group sessions, 3 times/week - 72 Meditation training (15–20 min after each exercise training session) - 4 Sleep guidance offline session every 1.5 months - 4 Dietary guidance every 1.5 months - 24 Health education: once a week for 6 months, in groups	29.3		Structured program		[41]
**The Heritage Study** (China)	*N* = 600 (60–80 years with MCI and modifiable lifestyle factors)	24	**Expected: 316 sessions** - 156 cognitive training sessions: 12 structured group guidance cognitive education workshops and 144 individualized cognitive training sessions (3 times/week during the first 12 months) - 144 home-based physical exercise sessions (about 3 times/week during the first 12 months) - 8 nutritional sessions: 6 group nutrition workshops and 2 targeted dietary instruction visits - 4 vascular risks monitoring visits - 4 group psychological counseling sessions	13.2		Mixed		[94]
**CHINA-IN-MUDI** (China)	*N* = 772 (60–80 years at risk)	24	**Expected: 104 sessions** - 104 weekly integrated multidomain on-site intervention (lasting 100 min), including group physical training, group cognitive training, medical monitoring, and nutrition and cardiovascular lecture and consultant. - Participants were encouraged to perform at least five weekly sessions at home, with a minimum of 20 min per session	4.3		Structured program		[95]
**J-MINT PRIME Tamba** (Japan)	*N* = 100 (65–85 years with DASC-21 between 22–30 points and MMSE ≥ 24)	18	**Expected: 78 interventions**, delivered as one intervention per week and comprising four domains: - Lifestyle-related diseases management - Physical exercise (90 min per week) - 15 Nutrition counseling (3 times in-person guidance and 12 times telephone follow-up)apple - Cognitive training (4 days a week)	4.3		Structured program		[15]
**Brain Boosters** (USA)	*N* = 112 (≥ 65 years with SCD)	6	**Expected: 15 sessions** - 15 two-hour classes with education about a variety of memory support strategies and healthy lifestyle behaviors, focusing on physical and cognitive activity and stress management - Support in adopting new behaviors and tracking set goals with the Electronic Memory and Management Aid (EMMA) digital application	2.5		Structured program		[96]
**The Gray Matters** (USA)	*N* = 104 (40–64 years, at-risk or with MCI)	6	**Expected: 39 sessions** - 39 MI educational sessions - Social engagement workbook - Smartphone application with tips, feedback and weekly summaries - Personal coach (28 students volunteered to be personal coaches, who provided a weekly email or text message to participants to provide emotional support and encouragement of lifestyle goals)	6.5		Structured program		[97]
**APPLE Tree programme** (UK)	*N* = 352 (≥ 60 years with SCD or MCI)	6	**Expected: 30 sessions** - 20 group sessions during the first 6 months: 10 1-hour group video call sessions every fortnight, and video-call ‘tea breaks’, less structured, to facilitate social sessions, in-between intervention sessions, with the same group - 10 individual goal‑setting phone calls after each main session (every 2 weeks) during the first 6 months - Access to an online cognitive training platform (the number of sessions is not specified)	5		Structured program		[98]
**BetterBrains** (Australia)	*N* = 645 (40–70 years, have a family history of dementia, and at least one modifiable risk factor for dementia)	12	**Expected: 58** - 6 education and coaching sessions via telehealth during the active intervention phase - Access to psychoeducation material about dementia risk reduction - 52 weekly notifications and reminders to check-in on their recommended intervention.	4.8		Self-guided		[99]
**HAPPI MIND** (Australia)	*N* = 250 (45–65 years with ≥ 2 potential dementia risk factors)	24	**Expected: 7** - 1 Individualized report with dementia risk based on ANU-ADRI questionnaire - 6 individualized dementia risk reduction motivational interview sessions over 24 months (face- to- face or via telephone or video telehealth) with a trained nurse. Interviews will include education on dementia and dementia risk reduction materials. - Access to the HAPPI-MIND app with support for self-management dementia risk factors at home and track progress against risk reduction goals	0.3		Self-guided		[100]

*The studies are sorted by status (completed*,* ongoing)*,* WW-FINGERS network membership (only for ongoing studies)*,* country and continent of origin*

^*1*^*The multimodal dose in structured intervention programs only considers the number of guided sessions*,* except for individual cognitive training sessions that are typically performed at participants’ home*

*AD = Alzheimer’s disease. ADAS-cog = Alzheimer disease assessment scale-cognitive. ANU-ADRI = Australian National University–Alzheimer Disease Risk Index. CAIDE = Cardiovascular Risk Factors*,* Aging*,* and Incidence of Dementia. CERAD-TS = Consortium to Establish a Registry for Alzheimer Disease neuropsychological battery*,* total score. CV = cardiovascular. CVD = cardiovascular disease. DASC-21 = the Dementia Assessment Sheet in Community based Integrated Care System-21 items. EGCG = epigallocatechin gallate. EMAs = ecological momentary assessments. GP = general practitioner. IADL = Instrumental Activities of Daily Living. MCI = Mild Cognitive Impairment. MMSE = Mini-Mental State Examination. MO = months. MoCA = Montreal Cognitive Assessment. NTB = neuropsychological test battery. RBANS = Repeatable Battery for the Assessment of Neuropsychological Status. SCD = Subjective Cognitive Decline. ZAVEN = Z-scores of Attention*,* Verbal fluency*,* and Episodic memory for Nondemented older adults*

**Fig. 1:**
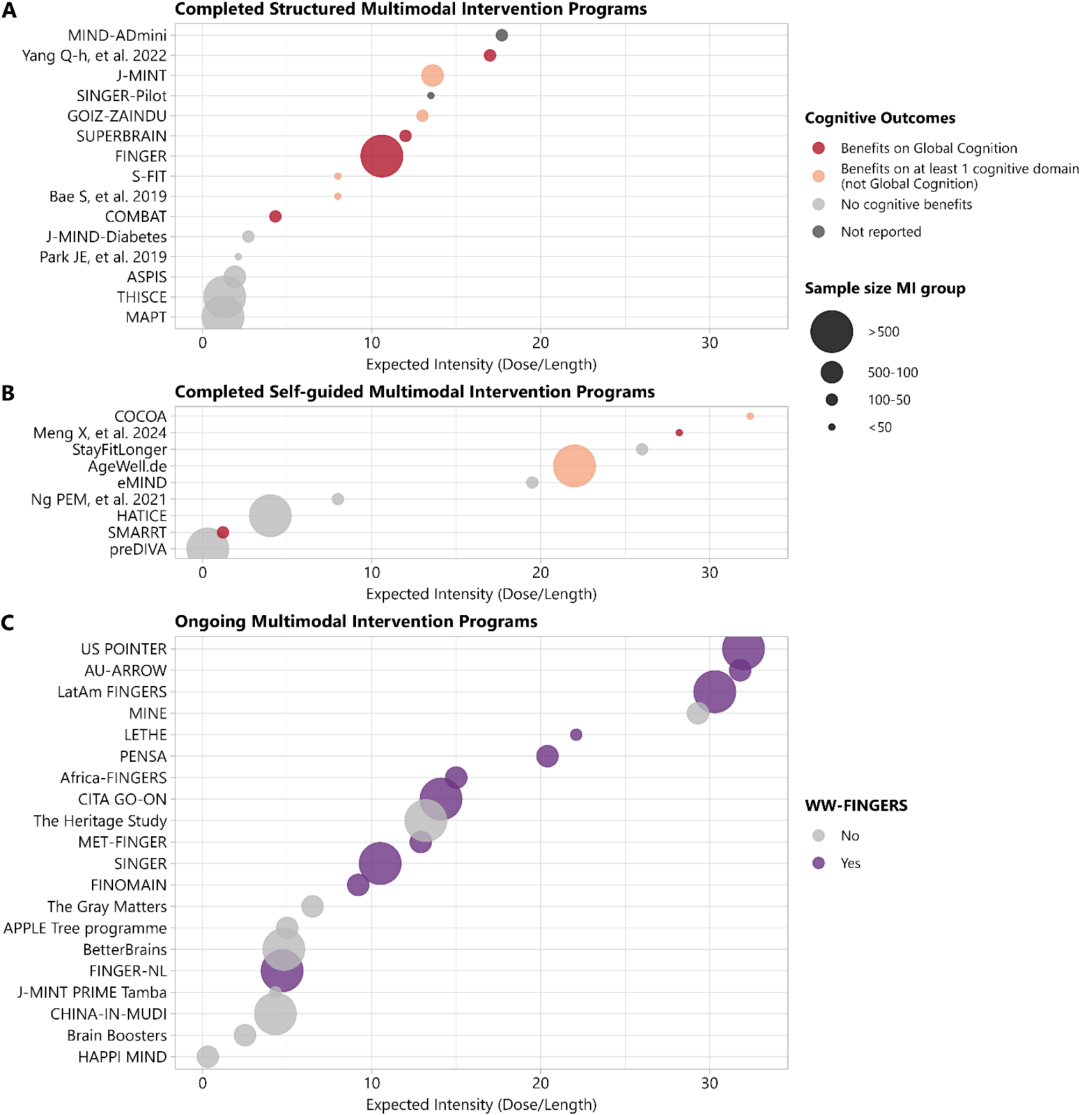
Intensity of multimodal intervention (MI) studies aimed at preventing cognitive decline, including (**A**) completed structured intervention programs (**A**, **B**) completed predominantly self-guided intervention programs, and (**C**) ongoing intervention programs categorized by WW-FINGERS network membership. Details of each study are provided in Table 2

As shown in Fig. 1A, structured multimodal intervention programs with expected intensities greater than 10 points are more likely to succeed in terms of improving cognitive performance or meeting primary cognitive outcomes. In turn, mainly self-guided or remote interventions should probably need greater intensities to impact cognition (Fig. 1B), because participants might be less likely to adhere, as evidenced by the AgeWell.de or HATICE results [25, 26]. On the other hand, ongoing studies are mainly framed within the WW-FINGERS network and thus follow the FINGER model of structured multimodal intervention, with expected intensities over 10 points (Fig. 1C). Specifically, the expected intensities ranged from 32 points in the US POINTER [27, 28], 31.8 points in the AU-ARROW [29], 30.3 points in the LatAm-FINGERs [30], 22.2 points in the LETHE trial [31], 20.4 points in the PENSA Study [32], 15.0 points in the Africa-FINGERS [33], 14.1 points in the CITA GO-ON [34], 12.9 points in the J-MINT and MET-FINGER trials [35, 36], 10.5 points in the SINGER [37], 9.2 points in the FINOMAIN [38], and 4.7 points in the FINGER-NL [39, 40]. Other ongoing multimodal intervention studies that are not members of the WW-FINGERS network usually have lower intensity scores ranging from 0.3 to 6.5 points, except for the MINE trial, which has an expected intensity of 29.3 points [41]. However, it is important to note that the intensity of the intervention is closely affected by the adherence and the content/quality of the intervention (although difficult to quantify). For instance, the observed intensity in the PENSA study after adjusting the expected dose by the average adherence to each intervention component was 14.5 points instead of 20.4 points (30% lower than expected by the design). Similarly, the observed intensity of the MAPT study was 33% lower than that expected by the design [9, 42], and this number was 36% in the GOIZ-ZAINDU study [18], and 55% in the HATICE study [26, 43]. However, the reporting of adherence or participation in intervention activities using cutoffs (e.g., percentage of participants with at least 66% adherence) prevents the assessment of the observed intensity, which requires adjusting the expected intensity by the average adherence to each intervention component. Moreover, information on the duration of cognitive or physical training sessions could allow a more accurate estimation of the intensity, as it could be equivalent to performing, for example, a 60-minute cognitive training session once a week and a 30-minute cognitive training session twice a week. Another aspect that could influence the estimation of the intensity is that the score proposed gives the same weight to all intervention components, although typically cognitive training or physical activity interventions have higher doses (e.g., weekly sessions) than nutrition or cardiovascular risk monitoring interventions (e.g., (bi)monthly visits).

While there is a strong rationale for delivering intensive, high-dose multimodal interventions to promote cognitive improvement or delay the onset of cognitive decline, it is equally crucial to address the challenge of achieving a sustained pattern of lifestyle modification. Striking a balance between intervention intensity, feasibility, cost-effectiveness, and long-term engagement is essential for the success and real-world applicability of these interventions. One potential approach involves a gradual increase in the intervention dose during the first 12–18 months, maintaining this heightened dose until the 2-year mark (in alignment with evidence of efficacy observed in the FINGER study), and thereafter gradually reducing the intervention dose so that the participants are likely to maintain the activities on their own after the intervention period is over. Another approach is to offer part of the program in a semistructured manner, for example, by using a hybrid intervention design. This approach is currently being tested in FINGER-NL, where part of the intervention program for all lifestyle domains is offered through a digital intervention platform. For example, online exercise instruction videos are made available with options for adapting the intensity of the work-out.

### Determinants of adherence

Understanding why individuals engage (or do not engage) in a particular behavior is vital in the context of behavior modification [44]. Moreover, the identification of determinants of adherence is linked to the mechanisms of an intervention as described in, for example, the Medical Research Council guidance regarding complex interventions [45]. Evidence suggests that baseline social and health conditions matter for adherence and efficacy, and interventions that consider psychosocial factors for engaging in a healthy lifestyle (e.g., motivation, environmental adjustment) may achieve better results [46].

To identify determinants of global adherence to multimodal interventions, we conducted a systematic search on PubMed (see details in the introduction section) and employed snowball methods, involving the pursuit of references within references and electronic citation tracking. Table 3 provides a summary of the evidence on the determinants of global adherence in multimodal interventions.

**Table 3:** Overview of determinants of global adherence to multimodal interventions identified in previous studies

Factors associated with poor adherence	Factors associated with good adherence
***Lifestyle factors*** - Current smoking status (9,14) ***Psychosocial factors*** - Depressive symptoms (9–11) - Lower health-related quality of life (9,11) - Higher Impact of memory problems on everyday life (12) - Higher perceived risk of Alzheimer’s disease (12) - Lower emotional stability (more anxious) (12) - ≥ 1 Fried frailty criteria (12) - Hopelessness (11) - Nonpositive perception of the study (11) ***Cardiovascular health*** - Diabetes (9,10,12) - Vascular risk (10) - Obesity, higher body mass index (9,12,14) - History of high blood pressure (9,14)	***Sociodemographic factors*** - Younger age (9,10,12) - Married (10) - Perceived social support (12) - Smaller town (< 200,000) (12) - Education (9) ***Cognition*** - Verbal memory (13) - Processing speed (10) - Global cognition (9,12) - Attention and working memory (9) ***Lifestyle factors*** - Healthier lifestyle behaviors (multi-lifestyle score) (10) - Physical activity (12) - Diet score (10) ***Psychosocial factors*** - Functionality (14)

In the recent years, numerous theories, frameworks, and models have emerged within the field of implementation science; however, their application in aging research has been limited [47]. An example of this is the Health Belief Model (HBM), which serves as a framework to explain and predict adherence to health and medical care recommendations. Its main premise lies in the notion that identifying beliefs and motivations related to health behaviors can inform the development of interventions aimed at increasing desirable health behaviors. This model defines key factors that explain health behaviors, including health knowledge, perceived susceptibility, perceived severity, perceived benefits of action, perceived barriers to action, cues to action, and self-efficacy. Notably, the HBM has been identified as the best-suited model for the development and evaluation of dementia prevention interventions [48]. In the context of the HBM, some studies have investigated the barriers and facilitators to participating in or implementing lifestyle interventions for dementia prevention using qualitative methods, targeting participants [49, 50], healthcare professionals [51] or the general public [52]. Experience with cognitive disorders (through family history or indirectly), motivated attitudes toward prevention and willingness to participate in a prevention trial were found to be facilitators, while beliefs that dementia is part of normal aging and not preventable were found to be barriers to participation. However, barriers to and facilitators of dementia prevention may differ, for example, between different socioeconomic groups, cultures, and genders [53]. Barriers and facilitators of overall participation in a trial may also differ from factors associated with adherence, and they can also differ between the intervention domains.

While HBM factors provide insight into the determinants of adherence, it is unlikely that a single intervention strategy can universally enhance adherence among all participants. The success of lifestyle interventions may depend upon tailoring interventions to the individual characteristics of participants. This is especially relevant in the context of multimodal interventions, which can be burdensome and not universally accepted. Participants may require different focuses on different domains, so tailoring is needed within the intervention. It has been proposed that some people (for example those who are frail or have a lower cognitive reserve) may benefit from higher dose of intervention, while for other people a lower dose might be sufficient [42]. Other factors related to the design of multimodal interventions such as the type, intensity and delivery method, or context (e.g., population, setting, community) may also influence adherence [9]. The adaptation of evidence-based programs to particular settings or populations is thus essential for maximizing their effectiveness [54]. Addressing this evidence-to-practice gap can be facilitated by incorporating implementation science approaches such as Intervention Mapping [55, 56]. These methodologies emphasize the evaluation of context and integrate social determinants of health in the development of interventions. Moreover, they involve program users (implementers, adopters, and maintainers) in the evaluation process [56]. The use of Intervention Mapping or similar methodologies in the design of dementia prevention studies can enhance their relevance in diverse populations. For instance, the LatAM-FINGERS and the AFRICA-FINGERS initiatives adopted the RE-AIM (reach, effectiveness, adoption, implementation, and maintenance) framework to assess a project’s effectiveness, feasibility, and sustainability [30, 33]. This approach has the potential to narrow the gap in dementia prevention research between low and middle-income countries and high-income countries [30, 33].

It is a matter of social justice that accessibility to health services is provided with the principle of equity. Populations with low socioeconomic status (SES) are known to be less likely to access health care and, more importantly, may be more likely to have less healthier lifestyles. Moreover, previous literature has shown a clear relationship between the prevalence of dementia and low SES, measured as annual income, educational level, occupation, and even neighborhood income levels [57]. Another important social determinant of health associated with dementia development is gender. Both a higher prevalence and incidence of dementia have been reported in women than in men, with two-thirds of individuals living with dementia and AD being women [58]. Risk factors associated with gender, which are all interrelated, range from lifestyle and psychosocial factors to cognitive reserve and may also affect participation in and adherence to multimodal interventions [59].

## Conclusions

Multimodal interventions for dementia prevention are currently in the trial phase, seeking to prove their efficacy across diverse contexts and target populations and in combination with pharmaceuticals or nutraceuticals. A significant challenge lies in determining the minimum required dose and duration of lifestyle intervention to impact cognition, maintain participant adherence and ensure cost-effectiveness. Ideally, the multimodal intervention dosage should be the minimum required to induce the desired lifestyle changes that, in turn, result in meaningful cognitive benefits [42]. However, it is currently unknown how many metabolic equivalents (METs) of physical activity, or minutes of cognitive training, or the degree of adherence to a healthy diet, are necessary to impact cognition. The room for improving cognition through multimodal interventions in older adults is also unclear, as it is generally assumed that cognition cannot improve ad infinitum, but there is a plateau in which a higher dose does not translate into more cognitive benefits [42, 60]. If this information was available, the intervention dose could be adjusted according to each participant’s characteristics. This could entail, for instance, providing fewer physical activity sessions to a participant who already meets physical activity recommendations at baseline; but increasing the number of sessions if this participant decreased the adherence to such recommendations during the follow-up. This information gap has led to standardizing the dose of lifestyle intervention for all participants. Consequently, the “dose” in multimodal interventions is typically established based on evidence of feasibility and efficacy gathered from prior single-domain or multimodal interventions, considering the necessity for sufficient intensity (as general lifestyle recommendations alone may not be enough to influence cognitive change) [61], while also ensuring that participant motivation is sustained in the long-run.

To better understand the bidirectional relationship between the intervention dose, intensity and adherence, we propose harmonizing the reporting of adherence across multimodal lifestyle-based intervention studies for cognitive decline/dementia prevention, including both participation in intervention activities (average participation in each component) and lifestyle change, using dementia risk scores such as the LIBRA index. The intensity of a multimodal intervention could then be estimated by leveraging the dose, length and observed adherence with each intervention component. Although this intensity score may not encompass all potentially relevant factors (e.g., population or contextual characteristics, intervention quality or duration of sessions), it serves as a preliminary attempt to quantitatively describe this aspect of multimodal interventions. Cross-trial comparisons will be easier across studies, e.g., WW-FINGERS network trials, and others, facilitating an understanding of how intervention observed dose and intensity influence efficacy.

The identification of determinants of adherence to multimodal intervention is important for informing the design and implementation of precision prevention interventions. This article provides an overview of the evidence on the determinants of adherence to multimodal interventions, emphasizing the need to gain knowledge on barriers and facilitators of enrollment, participation, and engagement. The incorporation of implementation science approaches such as Intervention Mapping could enhance the representativeness of the target population, especially including low SES groups, potentially benefiting the most from dementia prevention initiatives. As the field progresses, a commitment to standardized reporting and a nuanced understanding of adherence determinants will be pivotal for advancing dementia prevention research toward meaningful outcomes for diverse populations worldwide.

The topic of adherence may also be quite important to the people participating in these interventions. Additional work should be carried out to understand how this information about adherence should be communicated to participants and to complement the understanding of adherence to multimodal interventions from the perspective of the participants. This type of work could complement and enrich our understanding of adherence in the short and long term.

## Electronic supplementary material

Below is the link to the electronic supplementary material.

Supplementary Material 1

## Data Availability

No datasets were generated or analysed during the current study.
