# Supplementary material for: Adherence and intensity in multimodal lifestyle-based interventions for cognitive decline prevention: state-of-the-art and future directions
Source: Alzheimers Res Ther. 2025 Mar 17;17:61. doi: 10.1186/s13195-025-01691-0 (PMC11912746; doi:10.1186/s13195-025-01691-0)
Supplement: Supplementary file 1 — Supplementary Material 1 [file 13195_2025_1691_MOESM1_ESM.docx]

**Supplementary Material**


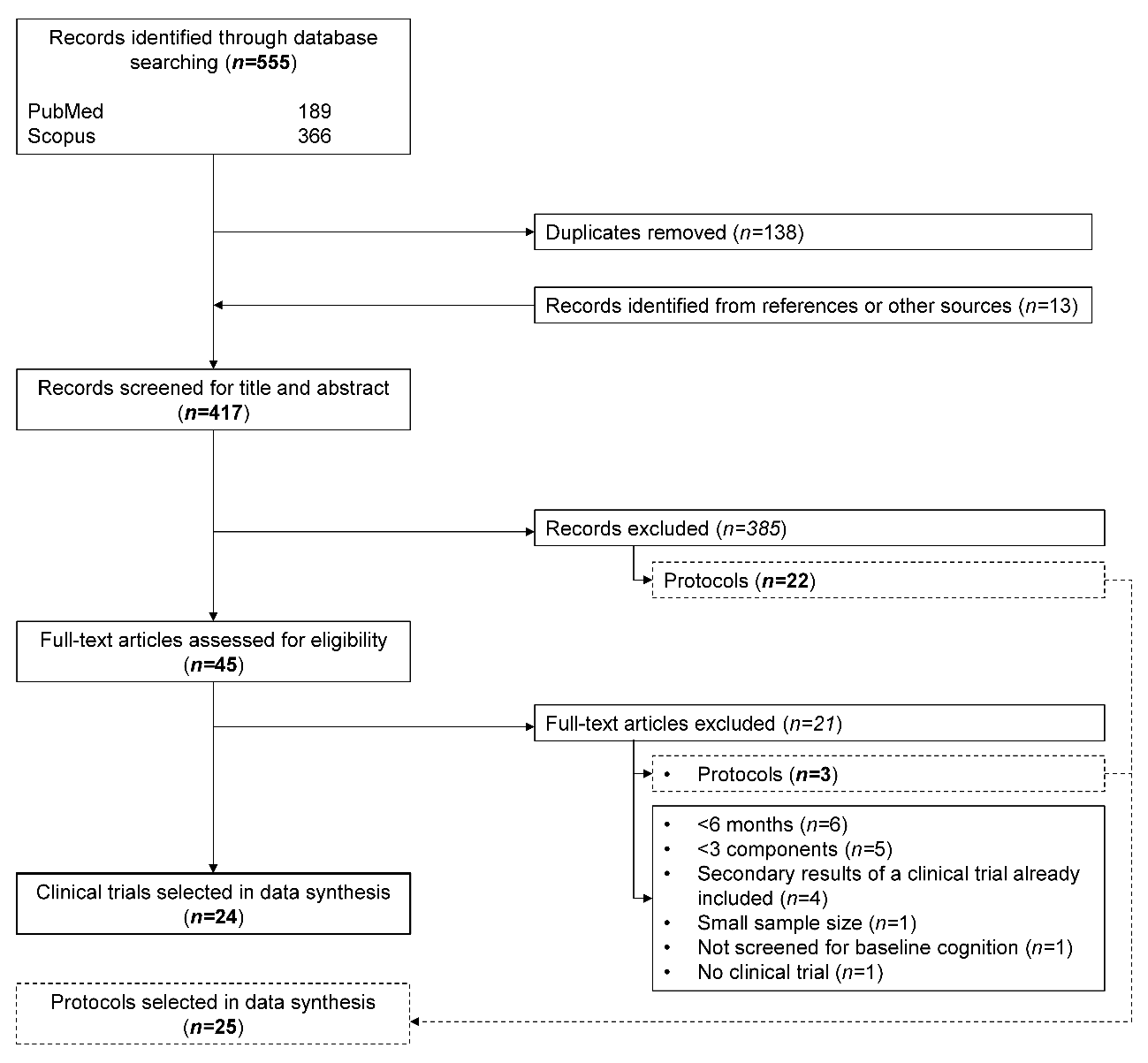


**Supplementary Figure 1.** PRISMA flow diagram illustrating the process of study selection.
